# Prevalence of HBV Infection and Knowledge of Hepatitis B Among Patients Attending Primary Care Clinics in Poland

**DOI:** 10.1007/s10900-015-0139-5

**Published:** 2015-12-23

**Authors:** Maria Ganczak, Gabriela Dmytrzyk-Daniłów, Marcin Korzeń, Marzena Drozd-Dąbrowska, Zbigniew Szych

**Affiliations:** Department of Public Health, Pomeranian Medical University, Żołnierska 48, 71-210 Szczecin, Poland; Vaccination Unit, Primary Care Clinic, Warszawska 30, 59-900 Zgorzelec, Poland; Faculty of Computer Science and Information Technology, West Pomeranian University of Technology, Żołnierska 49, 71-210 Szczecin, Poland; Primary Care Clinic, Parkowa 7, 74-100 Gryfino, Poland; Department of Computer Science and Education Quality Research, Pomeranian Medical University, Żołnierska 54, 71-210 Szczecin, Poland

**Keywords:** HBV, Knowledge, Prevalence, Vaccination, Primary care, Patients

## Abstract

It is well known that community awareness of hepatitis B (HB) can lead to vaccination and testing. The study objectives were to assess the prevalence of HBV infection and knowledge of HB among adult patients attending randomly selected primary care clinics. A cross-sectional sero-survey was conducted in March 2013 in the Zgorzelec region, Poland, with the use of an investigator-developed questionnaire containing 22 questions regarding HB knowledge. Serum samples were assayed for anti-HBc total and anti-HBs with enzyme immunoassay. The prevalence of anti-HBc total among 410 participants (median age 56 years) was 10.3 % (95 % CI 7.6–13.8 %), nobody was aware of an infection. The main sources of HB knowledge were the media and medical staff. The mean knowledge score was 14.8 ± 4.9; 76.7 % of the respondents had scores >50 %. Particular gaps were detected relating to knowledge of unprotected sexual intercourse and MTCT; 45.6 % patients were not aware of the potential asymptomatic course of HBV infection, 41.2 % about chronic HB treatment. A patient’s low educational level was negatively associated with a high knowledge level; the willingness for further education on HB and HBV vaccination in the past were independently associated with good knowledge. In conclusion, the HBV infection remains a public health threat in Poland, since the prevalence of infection markers in asymptomatic adult patients was high. Knowledge gaps call for awareness campaigns which may increase testing and diagnosis, audiences representing lower education level should be targeted first. Knowledge on HB might serve as an effective tool in decision making regarding vaccination.

## Introduction

Hepatitis B virus (HBV) infection remains a major global health problem affecting all countries, including Poland. Regarding current estimates, globally, approximately two billion people are infected with HBV, 248 million are HBsAg positive, with the seroprevalence 3.6 %. Of note, 780,000 persons die each year from hepatitis B (HB)—650,000 from cirrhosis and hepatocellular carcinoma due to chronic infection and another 130,000 from acute HB [1, 2].

In Poland there are approximately 350,000–450,000 carriers (0.5–1.5 %) of HBsAg in the population [3]. The peak incidence in Poland occurred during the 1980s and has been declining since. However, despite this decline, approximately 1000-1500 new cases still occur each year with the incidence of about 4.0/100,000 [4]. In 2013 acute cases accounted for 5.3 % of all HB cases and there has been a continuing downward trend in the last few years in both incidence and share of acute infection stage in the total number of cases. However, still 68 % of acute and 65 % of chronic infections were due to exposure during medical procedures [4]; such high percentages are not observed in other developed countries.

Therefore, despite the measures taken to break the spread of HBV infections at health care facilities, actions aimed at increasing the proportion of the population immunized through vaccination should be continued. In Poland the mandatory universal vaccination of children was launched in the early 1990s [5, 6]. Since that period active immunisation is also offered to recipients of blood and blood products, hemodialysed patients, household members and sexual partners of HBsAg carriers, health care workers and medical students [3, 5–7]. Efforts aimed at increasing community awareness and knowledge of HBV transmission and prevention should be of a special interest, especially among unvaccinated adults, not covered by the national immunisation program.

There is also concern that many of those infected have yet to be diagnosed [3–6]. There are several causes for this. HBV infection is often asymptomatic and can progress without any recognisable symptoms. Approximately 50–70 % of patients with acute HB have subclinical hepatitis and are unaware of the infection and of these, approximately 5–10 % will not clear the virus and remain chronically infected [8]. In order to increase case identification, population-wide education is essential so that individuals might recognise the infection and eventually come forward for treatment.

Therefore, this survey was planned to answer three research questions. Firstly, it was important to assess the prevalence of markers of HBV infection among adult patients from PCCs, born before the era of universal neonatal vaccination. This population was chosen for testing since identification, treatment, and also prevention in this group is paramount [3, 4]. According to previous epidemiological reports, they lived through the peak incidence years of HBV infection and would now be entering peak prevalence for severe complications [4, 8]. Of note, in 2013, in Poland chronic stages of HB were most commonly diagnosed in individuals aged 40–44 years [4]. Additionally, persons aged 65–74 years, which are common PCCs users, were found to be the main contributors regarding individuals with acute stages of HB in 2013. We believed that better understanding of prevalence among this cohort would be helpful not only on regarding individual patient perspective, but also for better predictions of the upcoming community burden.

Secondly, we wanted to evaluate the level of knowledge in this group regarding HB, i.e. modes of transmission, clinical symptoms, treatment and prevention. Such an assessment was considered to be significant with the aim to better understand possible obstacles regarding case finding as well as preventive efforts, while increasing receptiveness to the issue.

Individual knowledge might play a key role regarding vaccination uptake [9, 10], however this has not been thoroughly studied. Therefore, finally, the patient population was surveyed on their vaccination uptake to assess if knowledge on HB might be a determinant in decision making regarding vaccination.

## Methods

### Design and Setting

A cross-sectional sero-epidemiological survey was conducted in March 2013 among patients presented at randomly selected PCCs.

### Study Population and Sampling

The sampling frames included a complete list of public PCCs in the Zgorzelec region, in the south-western part of Poland, obtained from the local health department. All practices (for which there is 100 % access for the population) were stratified into urban and rural to ensure representation of different practice levels, with a random selection of 2 urban practices from the city of Zgorzelec (the capital of the region) and 1 rural practice.

### Study Instrument

A questionnaire which consisted of 33 closed questions was investigator-developed through an extensive literature review [9–11], and then administered by one of the researchers (G.D-D.). The content and convergent validity of the questionnaire was performed by an expert specializing in hepatology at the Pomeranian Medical University. It included questions that queried patients on the following:

- Demographic: age, gender, residency, literacy, socio-economic status (defined as low/medium/high), employment status
- Facility location
- Knowledge of HB: sources, self-assessment, knowledge items, self-willingness to improve knowledge
- History of previously diagnosed hepatitis
- History of HBV vaccination

Every person of at least 27 years of age (born before 1986, which was the year of universal neonatal vaccination) willing to participate was considered eligible.

A pilot study was carried out in one urban primary care center in Gryfino on 107 patients [12], the results have been included in the study. The authors’ choice regarding the relatively numerous group of patients involved in the pilot study was due to the fact that it was the first sero-survey conducted in Poland which queried PCCs patients on their HB knowledge and vaccination status not only with the use of questionnaires, but also by obtaining their blood samples. Therefore, the authors’ intention was to check different possible technical problems connected with sample collection and management [13].

Before answering the questions, respondents were asked to self-assess their knowledge on HB (4-gradient scaled questions; response categories ranged from “poor”, through “adequate” and “good” to “very good”).

Knowledge on HB was assessed by giving 1 point for each correct answer to the 22 items rated as “true”, “false”, “don’t’ know”, divided into 4 groups (clinical manifestation: symptoms, possible outcomes; transmission modes—real and myths; treatment; preventive measures). The scale measured knowledge from a minimum of 0 to maximum of 22. Scores for individuals were summed up to give a total knowledge score. Scores of 0–10 (<50 % of correct answers) were arbitrary taken as poor, 11–16 (50–75 % of correct answers)—as adequate knowledge of HB,  17–22 (more than 75 % of correct answers)—as good knowledge.

After answering the knowledge questions, respondents were asked to self-assess their willingness for further education regarding HB (response categories: “yes”, “no”, “don’t know”).

We defined a respondent as being immunised if reported at least two previous HBV vaccinations; this was checked in his/her vaccination card and sero-testing (the enzyme immunoassay for the quantitative detection of antibodies to the surface antigen of HBV—anti-HBs).

### Anti-HBc Testing

Enzyme immunoassay for the quality detection of antibody to the core antigen of HBV in human serum was used to detect anti-HBc (Hoffman-La Roche Ltd., Basel, Switzerland). At each facility blood samples were obtained from patients who gave informed written consent to participate. Testing was performed in two referential laboratories: in Szczecin (pilot group) and in Wrocław (study group). A code was given to the patient for the questionnaire and for the blood sample. 3 weeks after sampling the participants could call the investigators and obtain their results by stating their code.

The study received ethical approval from the Ethical Committee working under Medical Council Wrocław (1/DR/2013).

### Statistical Analysis

Data were analysed using a customized program STATISTICA PL Version 7.1. (StatSoft Inc., 2005). Our primary outcome variable was HB knowledge and we aimed to identify patient characteristics associated with this outcome (age, gender, residency, literacy, socio-economic and employment status, HBV vaccination, self-assessment of knowledge on HB, willingness to improve knowledge on HB). Besides descriptive analyses, categoric variables were compared (bivariate analyses) using the Chi square test with Yates’ correction and Fisher’s exact test, whilst the U Mann–Whitney test was used for numeric variables.

All variables significantly associated (*p* < 0.05) with “good knowledge” in the bivariate analysis were used to build a logistic regression model (i.e. the enter model was used), with the help of R software (R Development Core Team 2005) [14].

## Results

The pilot group did not differ significantly in demographic characteristics (*p* > 0.05) and vaccination uptake (*p* = 0.95) from the study group, therefore it was included to the analysis.

Of the total 413 consecutive patients eligible, 410 (99.3 %) consented to participate, 271 of them (66.1 %) were females. The median age for the study population was 56 years (range 27–85). Regarding literacy, 16.8 % (n = 69) had primary education, 28.3 % (n = 116)—vocational education, 37.1 % (n = 152) were high school graduates, 17.8 % (n = 73) had a university degree. Almost two-thirds (64.9 %; n = 266) of participants were living in urban areas. There were 13.4 % (n = 55) patients who described their socioeconomic status as high, 69.5 % (n = 285) as moderate, 17.1 % (n = 70) as low. Almost a half of the patients (47.1 %; n = 193) were employed, 31.7 % (n = 130) retired, 12.0 % (n = 49)—unemployed, the rest was supported financially by others. Most of the patents (81.7 %; n = 335) were from healthcare facilities located in urban areas.

### Anti-HBc Total Prevalence

Regarding patient history of previously diagnosed hepatitis, the answers were as follows: hepatitis C—2 respondents (0.5 %), hepatitis A—8 (2.0 %), 373 (91 %) ticked “no”; 27 (6.6 %)—“I don’t remember”.

Forty participants (9.8 %) refused to give blood for anti-HBc test. There was no statistical difference between responders and non-responders regarding demographic characteristic (data not shown). The prevalence of anti-HBc total in those who agreed was 10.3 % (38/370; 95 % CI 7.6–13.8 %). None of them had a history of clinical HB and none were aware of any infection.

### HBV Vaccine Uptake

More than a half of the participants, 55.4 % (227/410; 95 % CI 50.5–60.1 %), were previously vaccinated against HBV. The most commonly stated reason for immunization was the recommendation of referring surgeons (57.7 %); other reasons were media campaigns—10.1 %, the recommendations of GPs—4.8 %, or family/friends (4.0 %), a trip to HBV endemic countries—2.2 %; 21.1 % did not remember.

### Sources of HB Knowledge

Regarding sources of HB knowledge, 61.7 % of respondents received information from the media, 56.9 % from medical staff (doctors: 31.0 %, nurses 25.9 %), 20.7 % from friends, 16.6 % from brochures and leaflets and 10.0 % from the internet; this was a multiple choice question.

### Self-Assessment of HB Knowledge

Participants were asked to self-assess their knowledge; the answers were as follows: poor—58.5 % (240/410), adequate—27.3 % (112/410), good—11.0 % (45/410), very good—3.2 % (13/410).

### Overall Knowledge About HB

**Fig. 1:**
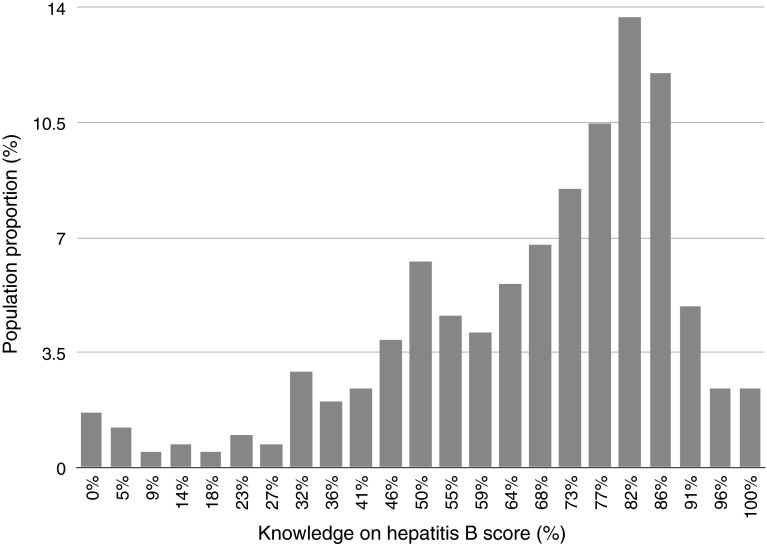
Proportion of the participants with particular scores regarding HB knowledge

The mean score of HB knowledge was 14.8 ± 4.9. More than three quarters (76.6 %) of the respondents scored >50 % of the correct answers. Figure 1 presents the proportion of the sample population with particular scores in the general HB knowledge section of the questionnaire. Regarding knowledge that HB is frequently asymptomatic, over a half (54.4 %) of respondents gave the correct answer (Table 1). Most participants knew about the possibile clinical outcomes of HBV infection: more than three quarters correctly selected chronic hepatitis, and liver cirrhosis, 69.5 %—liver cancer; 58.8 % ticked that there is a treatment for chronic HB.

**Table 1:** Knowledge on hepatitis B (all participants, vaccinated vs. not vaccinated); Zgorzelec region, Poland, n = 410

Statement	Correct answer	Category	Total	n	(%)	*p*
*Possible asymptomatic course of HBV infection*						
HBV infection is frequently asymptomatic	Yes	All respondents Vaccinated Not vaccinated	410 223 187	223 132 91	54.4 59.2 48.7	**0.04**
*Possibile clinical outcomes of HBV infection*						
Chronic HB	Yes	All respondents Vaccinated Not vaccinated	410 223 187	319 179 140	77.8 80.3 74.9	0.23
Liver cirrhosis	Yes	All respondents Vaccinated Not vaccinated	410 223 187	314 184 130	76.6 82.5 69.5	**0.003**
Hepatocellular carcinoma	Yes	All respondents Vaccinated Not vaccinated	410 223 187	285 162 123	69.5 72.6 65.8	0.16
*Routes of HBV transmission*						
Surgery	Yes	All respondents Vaccinated Not vaccinated	410 223 187	378 214 164	92.2 96.0 87.7	**0.004**
Endoscopy	Yes	All respondents Vaccinated Not vaccinated	410 223 187	243 150 93	59.3 67.3 49.7	**0.0005**
Hospitalisation other than surgery	Yes	All respondents Vaccinated Not vaccinated	410 223 187	207 122 85	50.5 54.7 45.5	**0.0005**
Dental procedures	Yes	All respondents Vaccinated Not vaccinated	410 223 187	358 206 152	87.3 92.4 81.3	**0.001**
Blood transfusions	Yes	All respondents Vaccinated Not vaccinated	410 223 187	358 200 158	87.3 89.7 84.5	0.15
Taking a blood sample	Yes	All respondents Vaccinated Not vaccinated	410 223 187	330 183 147	80.5 82.1 78.6	0.45
Mother-to-child transmission	Yes	All respondents Vaccinated Not vaccinated	410 223 187	205 126 79	50.0 56.5 42.2	**0.006**
Needle sharing	Yes	All respondents Vaccinated Not vaccinated	410 223 187	355 202 153	86.6 90.6 81.8	**0.01**
Unprotected sexual intercourse	Yes	All respondents Vaccinated Not vaccinated	410 223 187	222 122 98	54.1 54.7 52.4	0.71
Tattoos	Yes	All respondents Vaccinated Not vaccinated	410 223 187	355 202 153	86.6 90.6 81.8	**0.01**
Barber’s visit	Yes	All respondents Vaccinated Not vaccinated	410 223 187	257 158 99	62.7 70.9 52.9	**0.0003**
Cosmetic procedures	Yes	All respondents Vaccinated Not vaccinated	410 223 187	326 186 140	79.5 83.4 74.9	**0.04**
*Myths on HBV transmission*						
Mosquito bite	No	All respondents Vaccinated Not vaccinated	410 223 187	119 76 43	29.0 34.1 23.0	**0.02**
Sharing a toilet	No	All respondents Vaccinated Not vaccinated	410 223 187	132 82 50	32.2 36.8 26.7	**0.04**
Sharing a cup	No	All respondents Vaccinated Not vaccinated	410 223 187	129 80 49	31.5 35.9 26.2	**0.04**
Donating blood	No	All respondents Vaccinated Not vaccinated	410 223 187	72 34 38	17.6 15.2 20.3	0.22
*Treatment of chronic HB*						
There is a treatment for chronic HB	Yes	All respondents Vaccinated Not vaccinated	410 223 187	241 139 102	58.8 62.3 54.5	0.14
*Protective measures to prevent HBV infection*						
There is a vaccine for HBV	Yes	All respondents Vaccinated Not vaccinated	410 223 187	370 221 149	90.2 99.1 79.7	**<0.0001**

Bold values are statistically significant (*p* < 0.05)

When asked about routes of HBV transmission the majority of patients correctly identified nosocomial transmission during an operation procedure, dental procedures, blood transfussion and at needle sharing. Unprotected sexual intercourse was recognised as a risk factor by 54.1 % of respondents, mother-to-child transmission (MTCT)—by 50.0 %. However, 82.4 % of patients identified blood donation as a route of HBV transmission, 71.0 %—mosquito bites, 68.5 %—sharing a cup, 67.8 %—a toilet seat. In the overall sample; 90.2 % recognised vaccination as a protective measure to prevent HBV infection.

### Willingness for Further Education Regarding HB

The willingness for further education regarding HB was expressed by 47.1 % (193/410) of patients, 22.7 % (93/410) did not express it, the rest did not know.

### Determinants of HB Knowledge

The mean of HB knowledge of those who self-assessed it as adequate/good/very good was significantly higher than in those who assessed their knowledge as poor (mean 16.9 ± 3.6 and 13.3 ± 5.1 respectively; *p* < 0.0001); it was also higher in those who expressed their willingness for further education when compared to those who did not (mean 15.8 ± 4.5 and mean 13.9 ± 5.0 respectively; *p* < 0.0001). In patients who self-assessed their knowledge as adequate/good/very good there were more individuals with good knowledge level when compared to those who assessed their knowledge as poor (113/170, 66.5 % vs. 79/240, 32.9 %; *p* < 0.0001). In those who expressed their willingness for further education there were more individuals with good HB knowledge when compared to those who did not (112/193, 58.0 % vs. 30/93, 32.3 %; *p* < 0.0001).

The mean of HB knowledge level was significantly higher in vaccinated patients (mean 15.5 ± 4.5) than among unvaccinated (mean 13.9 ± 5.2); *p* = 0.002. There were more individuals with good HB knowledge among vaccinated patients than those nonvaccinated (118/226, 52.2 % vs. 73/184, 39.7 %; *p* = 0.02). As presented in the Table 1, the differences in HB knowledge level were observed regarding the possible asymptomatic course of HBV infection, some clinical outcomes and routes of HBV transmission, and protective measures.

In patients with high education level (university/high school graduates) there were more individuals with good HB knowledge when compared to those with low education level (123/225, 54.7 % vs. 69/185, 37.3 % respectively; *p* = 0.0007). In employed patients there were more individuals with good HB knowledge when compared to those unemployed (100/193, 51.8 % vs. 15/49, 30.6 %; *p* = 0.01). In patients with good social status there were more individuals with good HB knowledge when compared to those with moderate and poor social status (35/55, 63.6 % vs. 133/285, 46.7 % and vs. 24/70, 34.3 %; *p* = 0.002 and *p* = 0.03 respectively).

No significant differences were found between HB knowledge level and age (*p* = 0.86), gender (*p* = 0.17), and residency (*p* > 0.69).

A multivariable regression model revealed that a patient’s low educational level was independently negatively associated with good knowledge (OR 0.30); Table 2. Furthermore, participants who self-assessed HB knowledge as low were less likely (OR 0.25) to have good knowledge. The willingness for further education on HB and HBV vaccination in the past were independently associated with good knowledge (OR 3.12 and OR 22.5 respectively).

**Table 2:** Determinants associated with good knowledge of hepatitis B; Zgorzelec region, Poland, 2012/13)

Determinants associated with good knowledge on HB	OR	95 % CI	*p*
Literacy: low	0.30	0.09–0.87	0.03
Self assessment of HB knowledge: low	0.25	0.11–0.56	<0.001
Educational need to expand knowledge on HB: yes	3.12	1.48–6.82	0.003
Being immunized for HBV: yes	22.50	4.75–44.78	0.003

## Discussion

### Overview of the Results

The survey evaluated the prevalence of HBV infection markers, knowledge of HB, and vaccination status among adult patients attending PCCs in the south-western region of Poland. One in ten participants had markers of HBV infection, none were aware of the fact; HBV vaccination uptake was 55 %. The main sources of information related to HB were medical personnel and media. The participants had a relatively good level of HB knowledge. Although most of respondents correctly identified some of the main transmission risks (the most widely known risk factors were surgical and dental procedures, as well as blood transfusion), knowledge on sexual and MTCT was disturbingly low; only one in two patients was aware of the possibile asymptomatic course of HBV infection. Some myths continued to exist regarding possible routes of HBV transmission. The results also revealed a negative association of low educational level and good HB knowledge. The vaccination for HBV in the past and the willingness for further education on HB were independently associated with good knowledge.

### Sources of Knowledge

Findings of other studies have shown that patients wish to personally discuss issues related to infections with their health providers, especially preventive methods, like vaccination [15]. Apart from the media, doctors and nurses were one of the main sources of knowledge about HB for patients, possibly due to specific risk factors presented in this population, including surgical procedures. This might explain the relatively good overall knowledge related to HB in this group; more than three quarters presented scores >50 %. The involvement of health care professionals regarding information on HB observed in this study points to them as an important chanel in knowledge delivery. Friends or relatives did not play a role as sources of information on HB for study participants. These results are not in line with the findings from a survey conducted in Pakistan, where, surprisingly, the primary source of information for patients with HB was through family, friends and neighbors [10]. However, the level of HB knowledge among that study cohort was low. Authors of other surveys also reported rather poor HB knowledge levels among study populations [10, 16–19]. As an example, scores >50 % were obtained by only one in six pregnant women surveyed in Cameroon [16], and by 42 % Turkish immigrants in the Netherlands [19].

### Knowledge on HB

The awareness of HB among patients is crucial as it may support the testing and further identification of those in need of treatment [20]. Our results showed that, despite the relatively high HB knowledge, almost a half of the studied population was unaware of the potential asymptomatic nature of HBV infection. Furthermore, more than 20 % of patients were not aware of any possibile clinical outcome, 41 % did not know that treatment of chronic HB is available. All this could create a barrier to coming forward for testing. This is similar to some earlier studies: the majority of pregnant women from antenatal clinics in Cameroon and of HBV infected patients from public hospitals in Pakistan did not know that a person infected with HBV is capable of being asymptomatic [10, 16]. In contrast, over 80 % of Chinese immigrants to British Columbia, Canada, knew that HBV can be spread by asymptomatic persons [21]. The lack of knowledge about possible clinical outcomes of HB among studied populations was also observed by others [9, 10]. Relatively meagre knowledge about sexual and the MTCT of HBV was observed among our participants which is in accordance with the findings reported on German population and on the general population of France [9, 22]. This might be a source of concern, as a lack of knowledge about the main routes of HBV transmission can negatively influence preventive measures and be attributed to a rise in HB frequency. Some myths still existed, like blood donation, mosquito bites, the sharing of common household items as possible routes of HBV transmission; this was also observed by other authors [9, 17, 21, 23].

One fact that may be of note, those who perceived self-knowledge as low had four times less chance to have good HB knowledge when compared to those who self-assessed their knowledge level as high. This finding might be of importance regarding HB education and management. Patients are able to correctly self-assess the knowledge level, however, it does not necessarily influence their willingness for further education in case the knowledge is low. The willingness for further education on HB was associated in this study with good, not low, knowledge level. It might be hypothesized that some people are generally more health-oriented than others, and therefore might be more sensitive to possible threats in terms of infections, including HBV, and also more motivated to deepen their knowledge [24]. Future research is needed to strengthen this hypothesis.

The HBV vaccination coverage in this study was not satisfactory, however, much higher than in the other countries [17–19, 21–23]. Detailed information regarding this issue was presented in our previous study [12]. Those unvaccinated against HBV had a significantly lower HB knowledge level when compared to those vaccinated; being vaccinated was related to more than a twenty times greater chance of good knowledge. However, the direction of this observed effect cannot be determined. It can be hypothesized that patients tend to seek information regarding this topic to make a decision in terms of immunisation and therefore were more knowledgeable on HB, or they wanted to know more about HB after vaccination. The first hypothesis is supported by results of other recent studies on HB knowledge which likewise revealed that participants who had undergone vaccination had significantly higher levels of HB knowledge [18, 25–27]. As the examples, knowledge was better among Chinese immigrants in Australia who had been vaccinated compared with those who were unvaccinated [27], it was also a significant factor related to vaccination (OR 6.7) among students in the Ivory Coast [18].

There could be another mechanism to explain a higher level of HB knowledge in those vaccinated for HBV, as well. As more than a half of vaccinated patients were immunised due to the request of surgeons referring them for various medical procedures, it can be also assumed that surgeons played a key role as a source for knowledge on HB for that group. Despite the mechanism of how the knowledge is gained, individual knowledge together with awareness on HB might serve as an effective tool in decision making regarding vaccination. It has been proven by others that health-educational interventions, regarding knowledge of HB and protective measures, could measurably increase acceptance of immunisation [28, 29].

Proper patient education can be effective but needs to target the right population group [20]. Multivariate analysis highlighted an association between educational level and HB knowledge in this study. This result is in line with current literature [16, 17, 22, 30] which reported that a higher level of qualification positively correlates with knowledge about HB. Since the prevalence of HB is higher in those in an economically and socially vulnerable situation [28], this finding underlines the necessity to reduce social inequalities in health [22]. Other authors found age, female gender, marriage, social status and living in rural areas were associated with better knowledge [16, 19, 22, 30].

## Limitations

Our results may be not be generalizable to PCCs located in other regions of the country, especially in towns of more than 40,000 inhabitants. Further studies at a national level would be of value. Secondly, the drop out for anti-HBs testing could introduce a self-selection bias. Finally, considering a cross-sectional design, it was not possibile to rule out or infer any cause-effect relationship between the factors assessed and HB knowledge. Nevertheless, the strength of the study was due to the administration of questionnaires through face-to-face interviews with patients from randomly selected PCCs with an excellent response rate of 98 %. Moreover, HBV infection and vaccination status was not only based on self-reports but also on the results of serological tests. We believe that our result is robust enough to confirm hypothesis that inadequate knowledge on HB is prevalent among the community, and this factor might play an important role in the high prevalence of HBV markers.

## Conclusions and Recommendations

Our study estimated that one in ten of asymptomatic adult patients from PCCs, presented a serological marker of HBV infection, which shows that in Poland HBV remains a serious public health threat on the community level. Furthermore, gaps in knowledge and existing myths call for educational actions which will result in a better protection against an infection and increase testing to detect those who need further diagnostic and treatment. Actions should be taken on a national as well as community level, with the involvement of PCC personnel which should be aware of specific risk factors existing in the population and on an individual level. It has been proven that individual knowledge on HB might serve as an effective tool in decision making regarding vaccination. If educational and health promotion efforts are to remain cost-effective, they should be designed and targeted firstly at those with lower education levels.
